# Obstetrical Society of London

**Published:** 1877-08

**Authors:** 


### MISCELLANEOUS.

### Obstetrical Society of London.

Meeting, May 2d, 1877. Charles West, M. D., F. R. C. P., President, in the Chair.

Dr. Robert Barnes showed, for Dr. Bernard, of Londonderry, an apparatus for facilitating the injection into the uterine cavity, after labor, of water or perchloride of iron. It is made of india-rubber, and consists of a band which passes over the operator's hand. It carries a tube on the palmar aspect, which divides into three branches, so as to distribute the injection. There are also numerous small holes to scatter the fluid. With this instrument the operator's hand is conscious of all that goes on.

Dr. BARNES also exhibited, for Dr. Scott, of Canada, a pessary for complete procidentia uteri. It was constructed of wire, covered with rubber. The upper part consisted of a loop, which was intended to rest behind the cervix. The stem curved backward, over the perineum, and was supported, like Cutter's pessary, by a band which passed posteriorly. It thus was able to yield with the movements of the body. He had used it in about half a dozen cases, and found it to answer well. The patient could place it herself. The instrument could be obtained from Messrs. Blaise or Weiss.

Dr. HEYWOOD SMITH asked if the instrument was put forward as a modification of Cutter's pessary.

Dr. BARNES replied that it was not so stated by the inventor, but it appeared to be similar in principle.

The President announced that Mr. Spencer Wells' paper on Uterine Tumors associated with Pregnancy, would be read at either the June or July meeting of the Society.

---

### ON A NEW MODE OF TREATING CERTAIN CASES OF RETROFLEXION OF THE UNIMPREGNATED UTERUS.

By JAMES BRAITHWAITE, M. D., Leeds.

The author remarked that the application of Hodge's pessary combined with the occasional use of the sound as a redresser, sufficed for most cases of retroflexion, but there were some in which it failed. In these the only plan we had hitherto had to fall back upon was the continuous use of an intra-uterine stem, This was objectionable, since it acted as a foreign body, and kept up hyperæmia. For such cases the plan of treatment described in the paper was advocated. It was deduced from the principle that to straighten a stick the most effectual means was to bend it in the opposite direction, hence, to straighten a retroflexed uterus, temporary anteflexion should be produced. As a preliminary measure, he dilated the os uterus with a sponge tent, which he preferred to one of laminaria. If it could not be introduced past the point of flexion, he passed the first tent up to that point, and on the following day passed a second tent beyond it. The effect was to soften the uterus, to render it easier to undo the flexion, and to make the uterus more tolerant of the instrument afterwards used. This consisted of a brass wire, eight or nine inches long, soft enough to be easily bent. For introduction into the uterus, the last two inches of the wire was covered with india-rubber tubing, which fitted it tightly, and was closed at the end. This uterine portion was limited by a button at its proximal extremity, about two inches from the end, so that it did not reach within a quarter of an inch of the fundus. It was bent into such a shape as to hold the uterus in anteflexion. It was then introduced with the concavity backward, and rotated, as in redressing the uterus with the sound. An air-ball pessary was afterwards threaded over the wire, passed up as far as the button, and distended sufficiently to hold the whole instrument in position. Finally, the external portion of the wire was bent backward over the perineum, so as to occupy the sulcus between the buttocks. The instrument could not escape, get out of position, or enter further into the uterus; and there was no risk of injury from movement of the patient. It should be worn for four days, during which the patient should remain in bed, and opium should be administered. On its removal a Hodge's pessary is put in position. The uterus being now large and soft, ergot is given in large doses at long intervals, and water-douches administered. The author considered that the uterus is more ready to contract, and thus maintain itself in normal shape, after the withdrawal of the stimulus of an intra-uterine instrument which has been used for a short time only. The plan of treatment was, however, only recommended for extreme cases.

The author had cured the retroflexion in four cases which he had treated in this manner. In two others it was tried, but had to be abandoned. In the first, pain was produced by the use of the tent. Slight metritis was set up by the application of the instrument. It had very soon to be withdrawn, and but imperfect success was attained. In the second case, the instrument was inadvertently introduced on the day when a period was due. Violent pain was caused, and the instrument was expelled. This showed that the uterus itself helped when stimulated to action.

Dr. Priestley then said that some years ago Dr. Moir, the then President of the Obstetrical Society of Edinburgh, had recommended the dilatation of the uterus by sponge tents in cases of persistent retroflexion of the unimpregnated uterus, and the insertion afterwards of an intra-uterine stem with an oval-shaped bulb half way up, corresponding to the bend in the retroflexion, in order to produce a thickening at that point. He could not say what results had been obtained by this method, but Dr. Braithwaite's plan was the same, so far as the dilatation of the uterine cavity was concerned, although the latter part of the process was different.

Dr. Barnes was slow to resort to internal means until external means had failed. Hodge's pessary rarely failed. All intra-uterine supports, however ingenious, must cause a certain amount of chafing; they also fix the uterus. The organ should not be fixed. When all the means had failed, intra-uterine supports might be tried. Before introducing a Hodge, the displacement should be reduced. The action of the Hodge was gentle and safe, and when the uterus was once restored it could be worn with perfect ease. The cure was as complete as with intra-uterine instruments, for in that case also recurrence might take place. Professor Wallace, of Philadelphia, had shown him intra-uterine pessaries made of compressed sponge over a watch-spring curved back. As the sponge softened, the spring was liberated, and gradually restored the uterus. No external support was used. The inventor gave extremely good cases of the result of this treatment, especially when the fundus was locked under the promontory of the sacrum.

Dr. Graily Hewitt thought the instrument ingenious, and carrying out a necessary principle of treatment of flexion—viz., flexion in the opposite direction. He had done this by means of the sound, passed from time to time, reversed, and so held in position for a quarter or half hour. This, with the introduction of a modified Hodge, and the prone or semi-prone position, usually proved successful even in obstinate cases. There were some cases where the difficulties of treatment were almost insuperable, owing to adhesion of the uterus posteriorly, thinning of the uterine wall at the point of flexion, the chronicity of the flexion, and the hardness of the uterine tissue. He preferred, if possible, the avoidance of intra-uterine stems in retroflexion. Their difficulties and dangers were not light, and he used them only to a *very* limited extent. But if any intra-uterine treatment were to be adopted, Dr. Braithwaite's plan appeared admirable, especially his method of previously dilating the uterus. It should be recognized that there were limits to the possibility of cure, and that no permanent cure could be expected if there were much attenuation of the posterior uterine wall. In such cases the mere unbending of the uterus gave much distress. Another class of incurable cases was that of very old retroflexions in old women with rigidity of the uterus. Such difficult cases, in which it was sometimes necessary to relinquish the attempt at cure, were no doubt very rare. They often obtained much alleviation from Hodge's pessary.

Dr. Routh said the instrument presented many points of ingenuity. Some of them were not new, such as that the pessary was not of the full length of the uterus, and by this means irritation of the fundus was avoided. Dilatation by tent he had practiced for many years, but instead of using sponge tents, he employed laminaria, bent into the shape of the uterine cavity. This instrument fastened behind like Dr. Priestley's, and unlike Sir James Simpson's. The ball in the stem was new. He thought that the instrument would not answer in cases of soft uteri, which turned sometimes one way sometimes another; and that it was very extraordinary that a cure should be effected in four days. The discharge and irritations produced by an intra-uterine stem was sometimes most efficacious in curing, even when it went so far as to set up cellulitis. The thinning of the uterine wall was by that means remedied. Dr. Muirhead's pessary acted in the same way, for it produced in his hands much inflammation. It was desirable to set up a certain amount. He would like to know what was the previous duration of the four cases, and whether they remained cured.

Dr. Meadows said that younger members of the profession must be puzzled when experiences differed so widely. This subject was often discussed, and differences of opinion appeared to be on the increase. The discussion to-night would not allay the difference. He himself was quite opposed to Dr. Barnes and Dr. Graily Hewitt. The latter said that the thinning of the uterine wall was on the inside of the curve. He held exactly the opposite. When an india-rubber tube was bent, it was thinned on the convex side, and it was the same with the uterus. In a few cases which he had seen post-mortem, this was the case. There was a good instance of it in the Middlesex Hospital Museum. He was opposed to Dr. Barnes as to the use of an intra-uterine stem. He constantly found Hodge's pessary to fail, and not unfrequently to be injurious. If a sound were used after the Hodge was placed, the fundus would be found to be on the top of the Hodge. The views of displacement were too mechanical. Of mechanical means, the intra-uterine stem was far the most efficacious, and not mischievous, if of the proper kind. He often used it, and rarely saw any mischief from it, even in retroflexion. He would be sorry to endorse Dr. Routh's opinion that the production of slight inflammation is beneficial. This would be doing evil that good may come.

Dr. Bantock was surprised to find that retroflexion was still treated by means of a Hodge. The instrument was of use in retroversion, but of no use in retroflexion. A stem alone could effect a cure. Stems did not give rise to uterine contraction or hypertrophy. The effect of introducing a small bougie into the uterus was to cause relaxation, so that a larger one could be introduced next day. Retroversion with anteflexion was very difficult of cure, and cure could be effected by stems alone. He related an obstinate case which he had successfully cured. He first used one of his own stems with a Hodge's pessary. The fundus toppled over the posterior limb of the Hodge, and the stem slipped out by the next day. He then used Meadows' compound stem, in which a stem is fixed by an india-rubber band at right angles to a frame. This kept the uterus in admirable position. After three months' wear it was taken out, and the patient allowed to marry. She was now three months' pregnant.

Dr. Galabin asked whether Dr. Braithwaite had tried a modification of his instrument in cases of anteflexion? His own experience was, like that of Dr. Barnes and Dr. Graily Hewitt, that cases of retroflexion were very rare in which perseverance with Hodge's pessary, with the occasional use of the sound, was of no avail. But the treatment of anteflexion by vaginal pessaries was much less satisfactory. One reason of this was that, when a retroflexed uterus has once been restored, the pressure of the intestines comes to act, as it normally does, more on the posterior than the anterior surface of the uterus, since there is more room for them behind the uterus than in front. In this way the crevix being drawn backward by Hodge's pessary, a natural force of a gentle and continuous kind was called into play for the cure of retroflexion, to which there was nothing analogous in cases of anteflexion.

Dr. Rogers said that although there was much difference of opinion, all must admit that Hodge's pessary sometimes failed. He himself found it do so frequently. He had used intra-uterine stems extensively, and with the best results, first applying leeches, if necessary. It was not the practice at the Samaritan Hospital to set up inflammation intentionally. He had found elastic stem pessaries of much use in many cases.

Dr. Heywood Smith agreed with Dr. Meadows in his advocacy of constitutional treatment as of more importance than mechanical. The latter should generally be preceded by the use of leeches, or other depletory measures. Hence there were more failures among the out-patients of hospitals than among in-patients or in private practice, because the out-patients could not be put into a proper condition for treatment, nor could they obtain sufficient rest.

Dr. E. J. Hicks said that opinions as to the value of pessaries were very various, and there was no agreement even whether flexion produced any symptoms at all. General physicians said it did not. The flexion was not the only thing to be regarded, but the general state should also be treated. If local inflammation were excited by pessaries, the uterus was likely to be put into a worse condition.

Dr. Murray considered that pessaries were used much too frequently, and often in cases in which no discomfort arose from the flexion. He generally succeeded with Hodge's pessary, but in seven or eight cases had used intra-uterine stems. He had seen no ill effects from them, and had always been able to introduce them without dividing the os uteri, as some had advocated. The best form of stem was that of Dr. Wynn Williams.

Dr. Wynn Williams said he recommended stems in anteflexion, but not in retroflexion, and he had only once used one in the latter. This was a case which he had cured by one of his stems, supported by an india-rubber septum. The patient had been many months without benefit under the most eminent obstetricians of Dublin, and a Hodge's pessary could not be endured. A Hodge often failed because it was not long enough to reach the fundus. Before placing it, he restored the uterus with the sound, and passed the pessary over the handle. Before the sound was withdrawn the patient was made to cough and move, to insure it not being displaced afterwards. At first the Hodge should be as large as could be worn.

Dr. Wiltshire said that every one would be astonished at Dr. Bantock's statement that Hodge's pessary was useless in retroflexion. It was most useful in such cases. The intra-uterine stem was effective, and useful for very rare cases, but was an exceedingly dangerous instrument. Great benefit often resulted from simple restoration by the sound, combined with the use of the prone position, without any pessary.

Dr. Beigel said that pathologists paid little regard to female genital organs, and obstetricians were generally debarred from pathology. Hence arose great disadvantage to the science of flexions. He had recently examined about 500 uteri, and was surprised to find how little was known as to their pathology, and how little of what was stated in books was true. Of flexion he had only found ten or twelve cases in the 500. In these there was no change whatever of the uterine walls, even as seen by the microscope, and certainly no thinning of either side. Sometimes the sound could not be introduced, because the lining membrane was glued by inflammation. Nothing was so prejudicial as to set up even the *smallest amount* of inflammation. No one could tell where it would stop, or where it would draw the uterus. Any irritation within might set up by sympathy peri-uterine inflammation. It was very rare to find post mortem the genital organs perfectly normal. Signs of old plastic inflammation, especially about the tubes, were extremely common. As to treatment, he could not understand how a vaginal pessary could benefit a flexion. The uterus required very great power to straighten it; and the only means of cure, though one not always applicable, was an intra-uterine stem.

The President said that he spoke somewhat as representing the outsiders. It struck him as remarkable in what very different lights the same gentlemen regarded the uterus at different times. At one time all manner of sympathetic effects were attributed to it; at another it was considered more tolerant of treatment—not to say maltreatment—than any other organ. Sydenham truly said that it was the duty of those who practised medicine to find out indications for treatment rather than special remedies for this or that condition. The great point to aim at with regard to flexions of the uterus was to distinguish the different classes of these displacements and lay down rules when treatment should not be adopted because unnecessary or not beneficial. Great ingenuity had been shown in devising mechanical contrivances, but this might lead to mischievous practice. It should be remembered that the society should lead the medical profession in a particular branch. The result of Dr. Braithwaite's work was hardly such as to be encouraging. In one of his cases the uterus righted itself spontaneously, in two the treatment failed, in one the dilatation by tents could not be borne. And to gain this result the patient was subjected to very severe suffering, lying on her side for four days, and passing her urine into a sponge. It should be an extreme case to lead us to adopt such a treatment. Some cases of flexion needed no treatment; in a second class the suffering was from congestion, and was relieved by the cure of this; a third class, including especially cases where adhesions existed, would not bear treatment. Madame Boivin had written a tract on the frequency of a retroflexed uterus bound by adhesion as a cause of repeated abortion. Other flexions depended on the presence of fibroids, and in these the congestion should be chiefly treated. Virchow showed that thinning of the uterine wall took place on the concave side of the flexion, and brief treatment could not cure such a condition. Then a condition found so rarely post mortem as flexion is, according to Dr. Beigel's observation, could not be of very great importance. Stems were objectionable because of the risk of inflammation. The great principle of treatment should be, not to do harm if good could not be done. The effect of using stems might be like setting a house on fire in order to discover a treasure within it.

Dr. Braithwaite stated that the treatment described by him should be followed in exceptional cases only. Hodge's pessary was usually efficient, but in some rare cases it failed, and recourse must be had to stems. Cure was not effected in four days, but the uterus was placed in position for recovery, and the application of a Hodge then became effective.—*Obstetrical Journal.*

